# Multifocal streptococcus intermedius abscess mimicking neurocysticercosis clinical and radiological findings: A case report and literature review

**DOI:** 10.1016/j.radcr.2025.12.056

**Published:** 2026-02-10

**Authors:** Shuang Xu, Dylan Khoo, Jorn Van der veken, Jamie Miller, Marc Agzarian, Jie Ding

**Affiliations:** a Department of neurosurgery, Royal Adelaide Hospital, Port Rd, Adelaide, South Australia, 5000, Australia; b The University of Adelaide, Port Road, Adelaide, South Australia, 5000, Australia; c Department of Medicine, Royal Adelaide Hospital, Port Road, Adelaide, South Australia, 5000, Australia; d South Australia Medical Imaging, Flinders Medical Centre, Flinders dr, Bedford Park, South Australia, 5042, Australia; e Flinders University, College of Medicine and Public Health, Flinders Health and Medical Research Institute, Sturt Rd, Bedford Park, South Australia, 5042, Australia; f Jones Radiology, 337 South Terrace, Adelaide, South Australia, 5000, Australia

**Keywords:** Streptococcus intermedius, Multifocal cerebral abscess, Neurocysticercosis, Magnetic resonance imaging

## Abstract

Streptococcus intermedius is an opportunistic pathogen capable of causing rapidly progressive, life-threatening cerebral abscesses. Diagnosis can be difficult because clinical features are nonspecific, cerebrospinal fluid findings may be negative, and biopsy results take time. Neuroimaging therefore plays a crucial role. While most reported *S. intermedius* abscesses present as a single lesion, we describe an immunocompetent young man with unusual multifocal brain involvement initially mistaken for neurocysticercosis. A 33-year-old male presented with fever, headache, and rapidly progressive confusion. CT brain was unremarkable, whereas MRI demonstrated multiple ring-enhancing lesions with marked diffusion restriction across both hemispheres, the brainstem, and cerebellum. These were first interpreted as neurocysticercosis; however, stereotactic biopsy confirmed *S. intermedius*. Retrospective MRI review showed features favouring pyogenic abscess—uniform profound restricted diffusion, peripheral susceptibility from haemorrhage, and absence of a scolex. The patient was already receiving ceftriaxone for pneumonia, which also covers *S. intermedius*, and improved clinically with subsequent radiological resolution. This case highlights that *S. intermedius* abscesses may occur in immunocompetent hosts and closely mimic parasitic infection. Careful evaluation of MRI characteristics and early biopsy are essential for correct diagnosis. Prompt treatment with ceftriaxone and metronidazole can be lifesaving, and increased awareness may prevent future misdiagnosis.

## Introduction

Cerebral abscess is a focal infection characterised by a collection of pus in a walled off fibrotic capsule. They are commonly caused by bacteria, but can also be caused by fungal, mycobacterial, or parasitic pathogens. Although relatively rare, with an estimated annual incidence of 0.3 to 1.3 per 100,000 individuals, they represent a significant cause of morbidity and mortality due to potential neurological sequelae [[1], [2], [3]]. Among the various pathogens responsible for brain abscesses, *Streptococcus intermedius* is commonly implicated, which belongs to the *Streptococcus anginosus* group (also known as the *Streptococcus milleri* group) along with *S. anginosus* and *S. consellatus [*4]. *S. anginosus* species are gram positive, catalase-negative facultative anaerobic cocci that are a part of normal human flora and colonise the oral cavity and gastrointestinal tract [5]. Typically, this organism is an opportunistic pathogen, predominantly affecting immunocompromised individuals, such as those with diabetes, malignancies, malnutrition, or acquired immune deficiency syndrome [6]. The clinical presentation of these infections is often nonspecific, including fever, lethargy, headache, and confusion which can complicate timely diagnosis. *S. intermedius* cerebral abscesses typically demonstrate increased white blood count and/or protein, and reduced glucose in cerebrospinal fluid (CSF). Along with their characteristic appearance on magnetic resonance imaging (MRI) showing a ring-enhancing lesion with uniform profound restricted diffusion and surrounding oedema, this enables early definitive diagnosis and the timely initiation of antibiotic therapy [7,8]. Cerebral abscesses usually present as solitary mass lesions, [9] although rare multifocal cases of *S. intermedius* have been described. Consequently, when multifocal lesions appear on imaging combined with negative cerebrospinal fluid results, the diagnosis of *S. intermedius* abscesses may not be suggested. In this report, we present a rare case of a 33-year-old immunocompetent male who developed multifocal cerebral abscesses due to *S. intermedius.* Initially, the radiological findings were suggestive of neurocysticercosis, but subsequent biopsy confirmed the presence of S. intermedius abscesses. Fortunately, the patient also had concurrent pneumonia, which prompted the early initiation of appropriate antibiotic therapy, leading to a favourable outcome.

## Case report

**Fig. 1:**
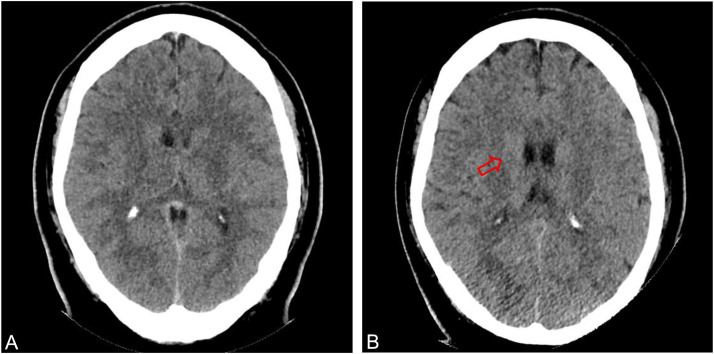
Axial Initial noncontrast CT brain (A) Two days after presentation axial postcontrast CT brain (B)- showing an enlarged right. Caudate head and ill-defined hypodensities in the right caudate and thalamus. Note that lack of contrast enhancement on the CT performed 2 days after presentation.

A 33-year-old previously healthy male presented to the Emergency Department with acute onset of severe confusion (Glasgow Coma Scale 12: E2V4M6), high-grade fever (39.1°C), headache, and productive cough. Other neurological examinations were unremarkable. Laboratory tests revealed marked leucocytosis (WBC 23.17 × 10⁹/L) and elevated C-reactive protein (101 mg/L). Chest X ray showed consolidation in the left lower lobe, while an initial noncontrast computed tomography (CT) brain was normal (Fig. 1A). He was diagnosed with community-acquired pneumonia and commenced on intravenous (IV) ceftriaxone and azithromycin.

**Fig. 2:**
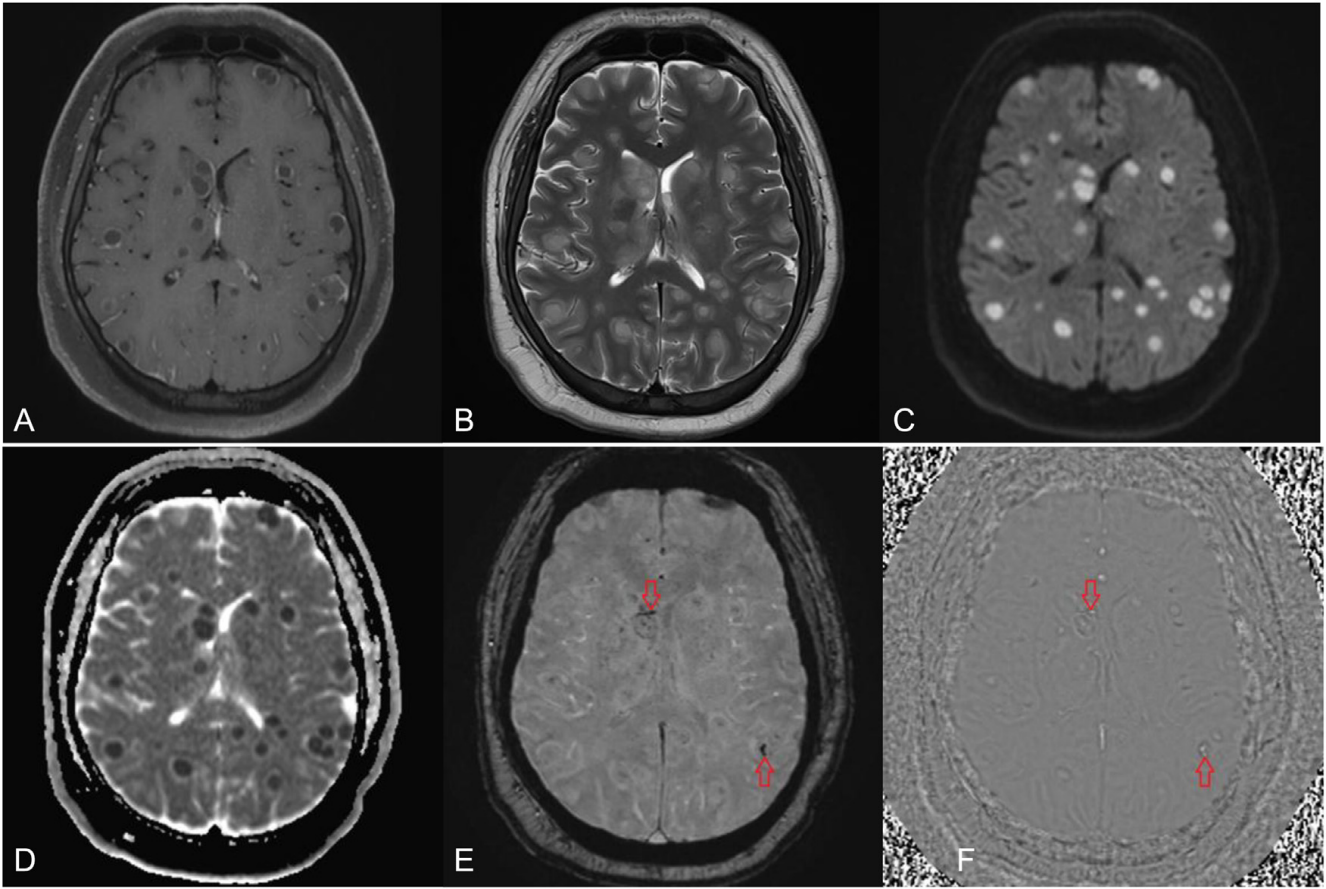
MRI head performed 2 days after presentation with axial postgadolinium contrast T1-weighted turbo spine echo image with fat saturation (A) showing multiple ring enhancing lesions. axial T2-weighted turbo spin echo (B) showing only mild lesional hyperintensity and minimal perilesional edema, axial diffusion-weighted imaging, DWI (C) and apparent diffusion coefficient, ADC map (D) showing profound, uniform restricted diffusion within all lesions (hyperintense on DWI and hypointense on ADC) and axial susceptibility-weighted imaging, SWI (E) and SWI filteredphase image (F); showing hemorrhagic foci (red arrows).

During hospitalization, his condition worsened: fever spiked to 40°C and GCS dropped to 8 (E1V2M5), necessitating ICU transfer. On day 3, repeat contrast CT brain revealed an enlarged right caudate head and ill-defined hypodensities in the right caudate and thalamus (Fig. 1B). Subsequent MRI brain revealed multifocal, round lesions scattered across the cerebral hemispheres, brainstem, and cerebellum. These were initially interpreted as consistent with neurocysticercosis (NCC) in the colloidal-vesicular stage; a pyogenic abscess was considered less likely due to multiplicity and lack of cerebritis (Fig. 2).

Lumbar puncture showed 10 polymorphonuclear cells, 3,500 mononuclear cells, 242,000 red blood cells, and no bacterial growth. Given the dilemma in diagnosis, consensus arrived at the need for tissue sampling for histology and culture. A right frontal craniotomy with intraoperative navigation was used to obtain a brain biopsy of the lesions and a small sample of surrounding cortex/white matter. Within a few days, cultures grew Streptococcus intermedius, sensitive to penicillin. Dexamethasone was administered to reduce cerebral edema, while ceftriaxone was continued.

**Fig. 3:**
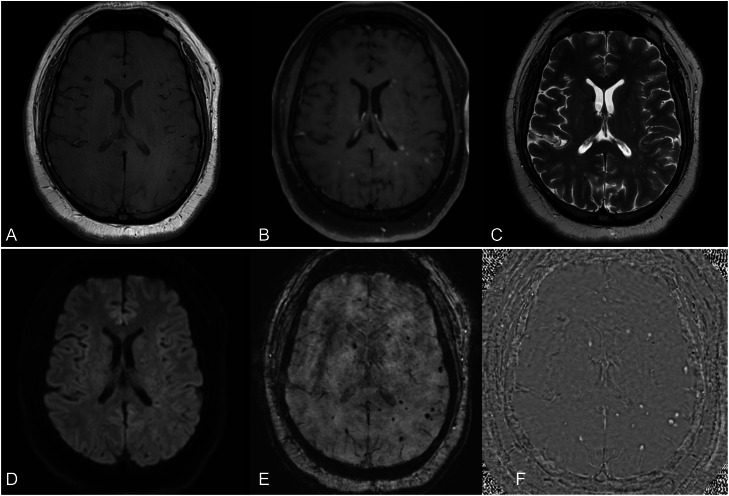
MRI head performed 3 months after presentation with axial pregadolinium contrast T1-weighted turbo spin echo image (A) & axial postgadolinium contrast T1-weighted turbo spin echo image with fat saturation (B) & axial T2-weighted turbo spine echo imaging (C), axial DWI (D), axial SWI (E) & SWI filtered phase image (F); showing a significant reduction in lesion size and resolution of perilesional edema with residual foci of hemorrhage.

The patient's neurological status improved spectacularly with steroid and antibiotic therapy—GCS returned to 15. He was stable to step down to the ward at day 13. Intravenous ceftriaxone was transitioned to oral amoxicillin, and steroids were tapered. The patient was subsequently transferred to a brain injury rehabilitation unit for 1 month for rehabilitation of cognitive functions and was later discharged home. Follow-up with infectious disease and MRI brain imaging was arranged with an MRI performed 3 months after presentation demonstrating reduced lesion size and resolution of perilesional oedema (Fig. 3) with complete clinical recovery.

## Discussion and literature review

The *S. anginosus* species can cause a wide spectrum of infectious disease, ranging from minor infections to bacteraemia and severe sepsis [10,11]. *S. intermedius* has propensity to cause pyogenic deep tissue infections and abscesses due to unique virulence factors, such as production of sialidase and hyaluronidase which destroys host tissues [12,13]. Furthermore, co-infection with anaerobic pathogens is common, especially when there is abscess formation [14,15].

*S. intermedius* has also been found to have an association with brain abscesses [16,17]. Often these are an isolated lesion, however, there have been cases of patients with multiple brain lesions [[18], [19], [20], [21]]. Brain abscesses occur either via contiguous spread from a parameningeal site of infection (eg, middle ear, mastoids, or sinuses) or via haematogenous spread from a distant focus of infection (eg, bacteraemia or septic emboli) [22].

In this case, given the multifocal brain lesions and absence of a contiguous source (XR OPG showed dental caries in 28 but did not reveal any dental abscesses), haematogenous spread would be the favoured pathogenesis with the patient’s pulmonary infection as the source. Although blood cultures were negative, this finding is consistent with the majority of *Streptococcus intermedius* brain abscess cases, in which cultures are often sterile and the organism is instead identified through direct sampling and analysis of purulent material [9,13]. This may be explained by a false-negative result due to insufficient blood culture volume or by transient bacteraemia that resolved spontaneously prior to sampling.

Given the clinical presentation is atypical, accurate diagnosis of *S. intermedius* brain abscesses relies on a combination of CSF analysis, MRI, and the gold-standard confirmation via biopsy and culture. It is important to note that the initial noncontrast CT head was normal, and the follow-up contrast-enhanced CT brain showed subtle abnormalities in stark comparison to the contemporaneous MRI brain, which reinforces the importance of performing an MRI brain in cases of suspected cerebral abscess. MRI characteristically reveals ring-enhancing lesions with a thick capsule, central uniform profound diffusion restriction, and surrounding vasogenic oedema. In this case, however, CSF results were nondiagnostic, underscoring the vital importance of imaging to guide clinical management prior to biopsy confirmation.

Radiologically, differentiating *S. intermedius abscesses* from NCC can be challenging. Because of the presence of multiple, uniform lesions, the initially favoured diagnosis was that of neurocysticercosis (NCC) in its colloidal vesicular stage. The imaging appearance of NCC is largely dependent on the evolutionary stage of the parasite, and the overlap with pyogenic abscesses can be substantial. On closer inspection, however, revealed blood products within some lesions on susceptibility weighted imaging (SWI) —an atypical finding for NCC. Moreover, all lesions demonstrated profound uniform restricted diffusion, a classic feature of pyogenic abscesses (Fig. 2). While colloidal vesicular NCC lesions can occasionally show homogeneous restricted diffusion, this is not their usual appearance. Crucially, no degenerating scolex was identified in any of the lesions, again arguing against NCC.

In the vesicular phase, NCC lesions typically exhibit a CSF-like signal without significant surrounding oedema. Occasionally, a punctate mural nodule corresponding to the scolex may be identified, and in some cases, diffusion-weighted imaging demonstrates small hyperintense foci at the site of the scolex. In the colloidal vesicular stage, lesions demonstrate ring enhancement after contrast administration, often with marked perilesional oedema reflecting the inflammatory host response [23]. Up to 20% of cases may exhibit an enhancing mural nodule representing the parasite, while a smaller proportion (∼5%) show homogeneous restricted DWI in the absence of a visible scolex. This stage is particularly challenging to differentiate from a bacterial abscess, given the overlapping features of ring enhancement and peri-lesional oedema [24].

It is important to note that lesion number is not a reliable discriminator between NCC and abscess. NCC can present as a solitary lesion, while *S. intermedius* abscesses, although classically solitary, may occasionally present with multiple lesions [25,26].

Similarly, the location of lesions is not pathognomonic. While the majority of NCC lesions are parenchymal (reported in 66.3% of cases, 263/342 lesions in 1 series), involvement of the ventricular system, brainstem, and subarachnoid spaces is well documented [23].

A distinguishing feature of NCC is the simultaneous presence of lesions in multiple evolutionary phases within the same patient. Identification of a scolex remains the most reliable diagnostic hallmark. On MRI, the scolex is highly conspicuous on DWI, manifesting as a central dot-like hyperintensity within the cyst. High resolution 3D T2 weighted MRI have been shown to further improve the conspicuity of intraventricular and cisternal cysts and aid in identifying the scolex [27].

Despite similar MRI presentation of *S. intermedius* and NCC, their treatments are vastly different. *S. anginosus* species, and by extension S. intermedius, are susceptible to all beta-lactam antibiotics [28,29]. Hence, the preferred treatment would be an antimicrobial such as intravenous ceftriaxone. The addition of an agent such as intravenous metronidazole to address any anaerobic pathogens should also be considered if abscesses are detected. This antimicrobial regimen is consistent with most recommendations from the literature for empiric antimicrobial treatment of brain abscesses, whilst acknowledging there is a scarcity of treatment guidelines [22].

In contrast, NCC is an infection of the central nervous system caused by the tapeworm Taenia solium. NCC manifests with characteristic cystic lesions on neuroimaging [30]. However, its clinical presentation can be diverse depending on the location of lesions, number of lesions (which can range from single to multiple), and degree of inflammatory response depending on the stage of the cystercerci [31,32]. Recommended management of NCC is with antiparasitic treatment (if viable cysts are identified), adjunct corticosteroids for anti-inflammatory effect, and antiepileptics (if there are seizures) [30,33].

In the present case, the patient fortunately received intravenous ceftriaxone for the concurrently diagnosed pneumonia, which also provided effective coverage for the cerebral abscesses—despite the initial misdiagnosis during the first few days of hospitalization.

## Conclusion

*Streptococcus intermedius* cerebral abscesses can occur in immunocompetent individuals and may present with negative CSF findings and unremarkable CT imaging. MRI brain should be performed in all suspected cases of cerebral abscess and careful interpretation is crucial as multifocal lesions may mimic neurocysticercosis. Histopathologic confirmation via biopsy and culture is the gold-standard and should be performed early.

Due to potential diagnostic ambiguity, early empiric antibiotic treatment—typically a beta-lactam such as ceftriaxone, often combined with metronidazole to address potential anaerobic co-infection—is recommended whilst awaiting biopsy results, as early treatment is crucial to optimize outcomes.

## Declaration of generative AI and AI-assisted technologies in the manuscript preparation process

During the preparation of this work the author(s) used Chatgpt 4.0 in order to check grammar. After using this tool/service, the author(s) reviewed and edited the content as needed and take(s) full responsibility for the content of the published article.

## Patient consent

Written informed consent was obtained from the patient for the publication of this case report and any accompanying images.

## Ethics approval and consent to participates

Not applicable.

## Availability of data and material

All available data is included for publication.

## Authors' contributions

SX: initial manuscript writing, patient consent, pictures selection and revision, manuscript revision, and submission.

DK: Contributed to manuscript writing, manuscript review and revision.

JV: manuscript review and supervision.

JM: manuscript review and revision.

MA: manuscript review and revision. Pictures selection and edition. Supervised the research and provided guidance throughout the study.

JD: manuscript review. pictures and manuscript edition.

All authors read and approved the final manuscript.
